# Choline Modulation of the A*β* P1-40 Channel Reconstituted into a Model Lipid Membrane

**DOI:** 10.4061/2010/752804

**Published:** 2011-01-03

**Authors:** Daniela Meleleo, Gabriella Notarachille, Silvia Micelli

**Affiliations:** Dipartimento Farmaco-Biologico, Università degli Studi di Bari, Via E. Orabona 4, 70126 Bari, Italy

## Abstract

Nicotinic acetylcholine receptors (AChRs), implicated in memory and learning, in subjects affected by Alzheimer's disease result altered. Stimulation of *α*7-nAChRs inhibits amyloid plaques and increases ACh release. *β*-amyloid peptide (A*β*P) forms ion channels in the cell and model phospholipid membranes that are retained responsible in Alzheimer disease. We tested if choline, precursor of ACh, could affect the A*β*P1-40 channels in oxidized cholesterol (OxCh) and in palmitoyl-oleoyl-phosphatidylcholine (POPC):Ch lipid bilayers. Choline concentrations of 5 × 10^−11^ M–1.5 × 10^−8^ M added to the *cis*- or *trans*-side of membrane quickly increased A*β*P1-40 ion channel frequency (events/min) and ion conductance in OxCh membranes, but not in POPC:Ch membranes. Circular Dichroism (CD) spectroscopy shows that after 24 and 48 hours of incubation with A*β*P1-40, choline stabilizes the random coil conformation of the peptide, making it less prone to fibrillate. These actions seem to be specific in that ACh is ineffective either in solution or on A*β*P1-40 channel incorporated into PLMs.

## 1. Introduction

Alzheimer's disease (AD) is an age-related neurodegenerative disorder that is characterized by a progressive loss of memory and deterioration of higher cognitive functions. The wealth of evidence of early and major synaptic damage and loss [1, 2], correlated with the severity of dementia [3–5], considers AD to be a disorder of synaptic function [6, 7]. 

Cholinergic neurons show particular vulnerability in the ageing process, which causes chronic deterioration of the brain component. It is a widespread belief among researchers that A*β*P peptides are involved in the loss of cholinergic neurons from the basal forebrain area in AD. Moreover, the mechanisms by which A*β*P peptides influence/cause degeneration of basal forebrain cholinergic neurons and/or the cognitive impairment characteristic of AD remain obscure, although there is little doubt that early synaptic pathology precedes the clinical symptoms of AD [8, 9].

Several hypotheses have been formulated to explain the onset of this disease such as: alterations in choline uptake, choline transport, impaired acetylcholine release, deficits in expression of nicotinic, and muscarinic receptors and channel formation by A*β*P oligomers. An increase in choline flux across the membranes of neuronal cells exposed to A*β*P has also been hypothesized to contribute to the selective vulnerability of cholinergic neurons in AD [10].

A*β*P is an amphiphilic peptide with a hydrophilic N-terminal domain (residues 1–28) and a hydrophobic C-terminal (residues 29–40 (−42)), the latter corresponding to a part of the transmembrane domain of APP. CD studies have shown that A*β*P changes its conformation from lipid-free A*β*P in order to bind to phospholipid vesicles. A*β*P consists of 48.9% random-coil, 23.5%  *β*-sheet, and 1.7% alpha-helix; while when reconstituted in phospholipid vesicles made up of 1,2-dimyristoyl-sn-glycero-3-phosphocholine, both *β*-sheet and *α*-helix contents increase by about 7%, that is, the *β*-sheet increased to 31.2% and the *α*-helix to 9.5%, respectively [11]. It has been demonstrated that A*β*P forms ion channels in the cell and model phospholipid membranes [12–14]; a *P*_*K*_^+^/*P*_Cl_^−^ permeability ratio of 11, with calcium permeability blocked by tromethamine and aluminium, has been reported [12]. Moreover, A*β*P disrupted calcium homeostasis and increased calcium intracellular concentrations; these events may be responsible for cellular toxicity [15]. Recent studies indicate that the peptide's ability to form ion channels depends on its conformational structure and on the peptide/lipid structure in the physiological environment. Some authors have found that membrane components, such as cholesterol (Ch) and gangliosides, alter the affinity of A*β*P for phospholipid membranes. In fact, Ch and gangliosides, once associated with phospholipid membranes, lead to an increase in *β*-sheet content and/or the rate of aggregation of A*β*P [16]. On the other hand, other authors have shown that, when A*β*P was added to a 33% Ch-containing 1,2-dimyristoyl-sn-glycero-3-phosphocholine vesicle, the structure of A*β*P was drastically altered, that is, the *β*-sheet structure decreased to zero while the *α*-helix increased to 58.8% [11]. In addition, alterations to the soluble Ch concentration and/or in Ch biosynthesis have been shown to affect the normal processing of APP, both in* vivo* and in *vitro *[17]. In a previous study, we investigated the role played by membrane composition on the interaction and self-assembly of A*β*P1-40 during pore formation in PLMs. This study showed that A*β*P1-40 has a higher propensity to form channels in OxCh PLM (as compared to other neutral phospholipid PLMs, whether they contain sterols or not), where the channels present high conductance, high frequency, anion selectivity, and long lifetime [14]. 

In this study, we investigated the effects of choline on the A*β*P1-40 ion channel in lipid bilayer membranes made up of OxCh and of POPC:Ch bilayer. OxCh membranes were used due to the evidence of a Ch-rich domain both in eukaryotic plasma membranes—such as brain and blood vessels—and in aged membrane plaque. Besides, A*β*P1-40 shows a higher affinity to Ch and its oxidation products than to phospholipids [14].

## 2. Materials and Methods

### 2.1. Single Channel Measurement

Channel activities were recorded in a lipid bilayer membrane made up of OxCh in n-decane (1:1, v:v) (Fluka) or POPC:Ch (65 : 35, w/w) in 1% n-decane. OxCh was obtained following the method of Tien et al. [18]. Bilayers were formed across a 300 *μ*m hole in a Teflon partition separating two Teflon chambers (volume 4000 *μ*l) which held symmetrical 50 mM KCl solutions, pH = 7, temperature 23 ± 1°C. The aqueous solutions were used unbuffered. The salts used in the experiments were of analytical grade. In all peptide experiments performed, the conductance and capacitance of each membrane was tested by applying a voltage of ±100 mV for 10–15 minutes under stirring to ensure that the membrane was stable. 

A stock solution of A*β*P1-40 (Sigma or EZBiolab) was prepared by dissolving A*β*P1-40 powder (0.1 mg) in 100 *μ*l of bidistilled sterile water under stirring for 3 minutes. From this solution, 5 *μ*l were withdrawn and diluted in 45 *μ*l of bidistilled sterile water under stirring for 3 minutes. Both solutions were stored at −20°C until use and 8.7 *μ*l of the second solution was added to the *cis*-side of the membrane, to obtain the final concentration of 5× 10^−8^ M. After A*β*P1-40 ion channel formation, few *μ*l of scalar dilution of choline (Fluka) stock solutions (1 × 10^−3^; 1 × 10^−5^; 1 × 10^−7^; 1 × 10^−8^ M) were added to obtain the final desired concentration on the *cis* or *trans* side of the Teflon chambers. The solutions were stirred after each addition of A*β*P1-40 or choline for 1 minute.

In single-channel experiments, the membrane current was monitored with an oscilloscope and recorded on a chart recorder for further data analysis by hand. The *cis* and *trans* chambers were connected to the amplifier head stage by Ag/AgCl electrodes in series with a voltage source and a highly sensitive current amplifier. The single-channel instrumentation had a time resolution of 1–10 msec depending on the magnitude of the single-channel conductance. The polarity of the voltage was defined according to the side where A*β*P was added (the *cis*-side). A *trans*-negative potential (indicated by a minus sign) means that a negative potential was applied to the *trans* side, the compartment opposite the one where A*β*P was added.

The phenomenology of A*β*P1-40 incorporation and choline action was studied as follows:

- to define the voltage-dependent characteristics of A*β*P1-40, we measured the amplitude of channel events at each membrane potential applied,
- to define the channel lifetime, from records extending over prolonged periods, the channel duration was measured considering the time between the opening and closing of each channel. The single channel data were obtained from at least two and sometimes four experiments (more than 100 single channels for each experiment) performed on different days. A histogram of channel conductance distribution for each experiment was constructed and fitted by a Gaussian distribution function (GraphPad Prism version 3.0; GraphPad Software Inc., http://www.graphpad.com/). Results are expressed as mean ± SE. The average lifetime of the conductance unit was estimated by the formula
  (1) $$N=A_{1}e^{\left(-t/\tau_{1}\right)}+A_{2}e^{\left(-t/\tau_{2}\right)}$$
  where *N* is the number of channels that remain open for a time equal to or greater than a certain time *t*, *A*_1_ and *A*_2_ are the zero time amplitudes, and *τ*_1_ and *τ*_2_ are related to the fast and slow components of the time constant, respectively. The single-exponential distribution is included in the formula (*A*_2_ = 0). To choose between the two models, we performed an appropriate statistical test (*F*-test, Graph Pad Prism version 3.0; Graph Pad Software, Inc, http://www.graphpad.com/),
- to identify the charge on the ion carrying the current, we measured the shift in the reversal potential induced by a change from a symmetrical to an asymmetrical KCl solution system. When the membrane conductance reached a virtually stable value, after addition of A*β*P1-40 to the* cis* chamber,we added choline to the* cis* or* trans* side. After stabilizing the membrane conductance, the KCl concentration was raised to 100 mM by adding concentrated salt solution to the *cis* side of the chamber.

The reversal potential was determined by changing the holding potential of ±4 mV step by step, and the potential at which the current was zero was taken as the reversal potential for the open channel.

The permeability ratio was calculated using the Goldman-Hodgkin-Katz equation

(2) $$V=\left(\frac{RT}{F}\right)*\ln\;\left\{\frac{\left(P_{K}{\left[K\right]}_{t}+P_{\text{Cl}}{\left[\text{Cl}\right]}_{c}\right)}{\left(P_{K}{\left[K\right]}_{c}+P_{\text{Cl}}{\left[\text{Cl}\right]}_{t}\right)}\right\},$$

where [*X*]_*t*_ and [*X*]_*c*_ are the concentrations of the ion species *X* in the *trans* and *cis* compartments, respectively and where *R*, *T* and *F* are molar gas constant, thermodynamic temperature and Faraday constant, respectively.

### 2.2. Circular Dichroism Measurement

CD spectra were recorded on a Jasco J-810 spectropolarimeter at 23 ± 1°C. Cells with a path length of 1 cm were used for spectra recorded between 200 and 260 nm, with sampling points every 0.5 nm. The A*β*P1-40 stock solution concentration was 5.77 × 10^−5^ M. The aqueous buffer used to dissolve the peptide was 50 mM KCl pH 7. Peptide samples were prepared from stock solution at a concentration of 5.77 × 10^−6^ M in 50 mM KCl pH 7. Samples containing choline were prepared by adding choline chloride at a concentration of 5.77 × 10^−9^ M or 5.77 × 10^−8^ M to the A*β*P1-40 samples, the same ratio (1 : 1000 or 1 : 100, resp.) of choline: A*β*P1-40 that was used in channel experiments. CD spectra were recorded 5 minutes, 24, and 48 hours after preparing the samples. Each CD spectrum consisted of five consecutive scans at a scanning speed of 20 nm/min. The samples were stored at 23 ± 1°C for subsequent analysis.

### 2.3. Choline Permeability across OxCh Membrane without and with A*β*P1-40 Channel Activity

Choline permeability was measured as follows: when the membrane turned “black” in the control experiments or when A*β*P1-40 manifested its channel activity and in the open state of the channel, 4 *μ*l of ethanol containing 4 *μ*Ci of the labelled choline ([^3^H]choline, Perkin-Elmer) were added to the *cis *side; the same amount of unlabeled choline solution was added to the opposite side. The solution was stirred mechanically. After 30 minutes, 10 *μ*l of fluid from both sides was collected. The radioactivity readings were performed with an LS 6500 Multipurpose Scintillation Counter (Beckman Coulter, USA) and the permeability coefficient calculated. Mean values ± SE of 3 experiments for each set of experiments are reported.

## 3. Results

### 3.1. Effect of Choline on A*β*P1-40 Channel Conductance

In this study, we evaluated the effect of choline on the A*β*P1-40 ion channel incorporated into PLMs made up of OxCh or POPC : Ch. 

First of all, in order to exclude any nonspecific and destabilizing effect of choline *per se *on PLMs used, we performed experiments by leaving choline in the medium facing the membrane for up to 24 hours. The stability of the PLM was tested by applying a voltage of ±100 mV for 10–15 minutes under stirring and monitoring constant values for conductance (25 pS) and capacitance (0.32 *μ*F/cm^2^); choline, over the range of concentrations used in the present work, caused no variations in membrane conductance and capacitance in bare membranes. 

**Figure 1:**
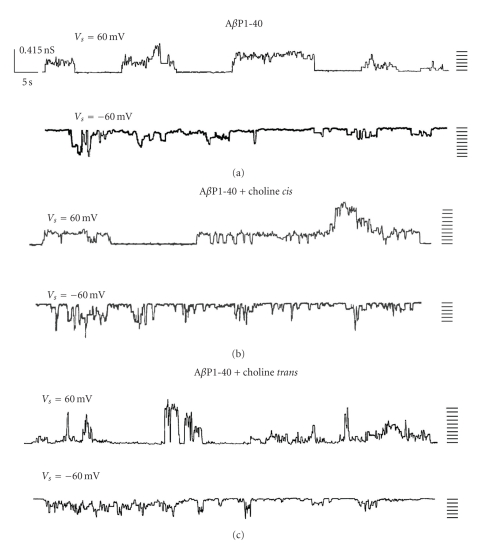
A*β*P1-40 channel activity in OxCh PLM in the absence and in the presence of choline. Representative A*β*P1-40 channel in the absence (a), in the presence of choline on the *cis* (b), or on the *trans* (c) side of the medium facing an OxCh PLM at an applied voltage of ±60 mV. Each trace represents a fragment of the recording of the activity obtained in individual experiments. Note the increased activity of the channel and the frequent multiple levels of conductance, especially at positive applied voltages, when choline is present. Experimental conditions KCl 50 mM, A*β*P1-40 5 × 10^−8^ M were present to the cis side of the membrane, choline 5 × 10^−11^ M was added on the cis or trans side of the membrane.

The incorporation of A*β*P1-40 (added to the *cis* side of the medium facing the membrane) into the lipid bilayer leads to nonrandom discrete square events that fluctuate between conductive and nonconductive states, compatible with channel-type openings, and closures with different conductance levels, lifetime and frequency. The pattern is similar to that found in our previous study [14], that is, we found higher conductance for the A*β*P1-40 channel incorporated into OxCh PLM.  Figure 1 shows examples of chart recordings of A*β*P1-40 channel formation in OxCh PLMs. 

**Figure 2:**
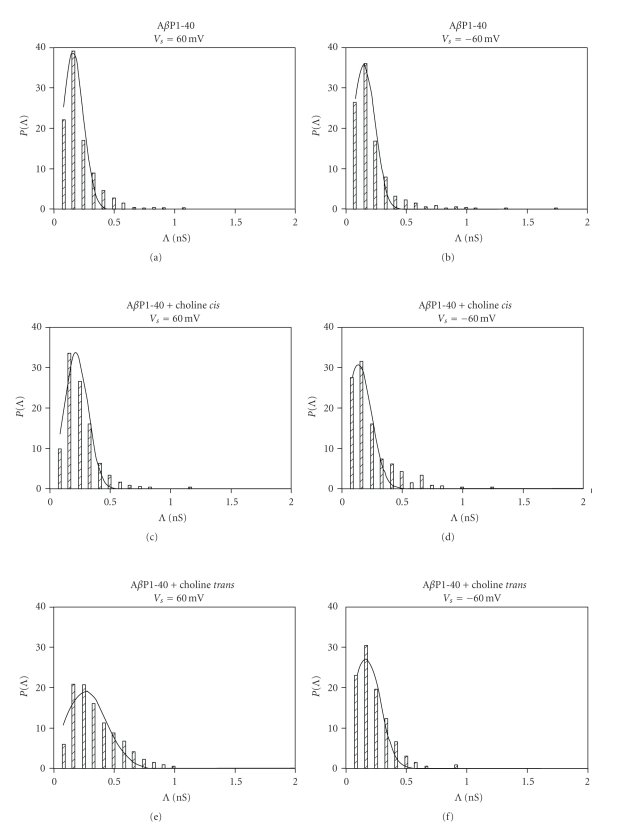
Amplitude histograms of A*β*P1-40 channel conductance. The histograms of the probability, PΛ, for the frequency of a given conductivity unit, relatives to each trace reported in Figure 1, were fitted by a Gaussian which is shown as a solid curve.

**Figure 3:**
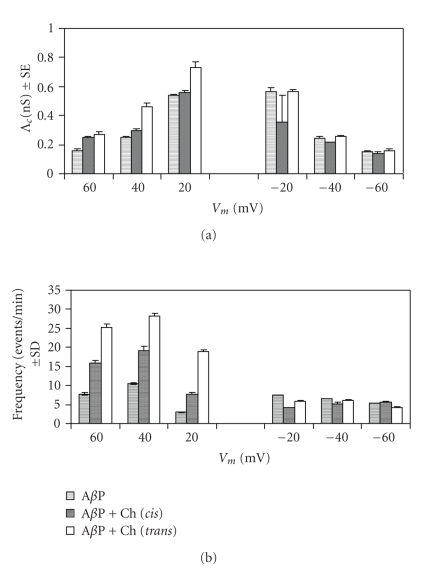
A*β*P1-40 channel mean conductance and frequency in OxCh PLM in the absence and in the presence of choline. (a) Λ_*c*_ ± SE and (b) frequency ± SD of A*β*P1-40 channel without and with choline (5 × 10^−11^ M) added to the *cis *or* trans* side of the chamber in OxCh PLM. The minimum and maximum number of channels considered (*N*) out of a total number of channels considered (*N*_*t*_) was: A*β*P1-40, 448 < *N* < 2080, *N*_*t*_ = 5493; A*β*P1-40 + choline *cis*, 115 < *N* < 898, *N*_*t*_ = 2269; A*β*P1-40 + choline *trans*, 448 < *N* < 948, *N*_*t*_ = 3844.

When the A*β*P1-40 channels fluctuated in the open state in OxCh PLMs, choline (5 × 10^−11^ M) was added to the *cis* or *trans* side of the medium facing the membrane. In a short time (about 5/30 minutes, *cis*/*trans*, resp.) choline determined an increase in A*β*P1-40 ion channel activity characterized by more frequent multiple levels of conductance. Furthermore, these patterns were more evident at positive applied voltages.  Figure 2 reports the histograms of conductance distribution for each experimental condition used in OxCh PLMs. All the histograms show single-peaked conductance distributions. The central value of conductance (Λ_*c*_) obtained by the Gaussian best-fit characterizes the conductance state of A*β*P1-40 channels in various membranes.  Figure 3(a) reports the central value of Λ_*c*_ ± SE at the different applied voltages for different experimental conditions in OxCh PLMs. In the presence of choline, no matter which side it is added, the Λ_c_ values are significantly higher at positive applied voltages than at negative ones (*P* < .0001 and *P* ≤ .0012 for choline added on the *cis* and on the *trans* side, resp.). If we compare the effect of choline on A*β*P1-40 channel conductance, it can be seen that choline added on the *trans* side increases channel conductance more than when added to the *cis* side.

**Figure 4:**
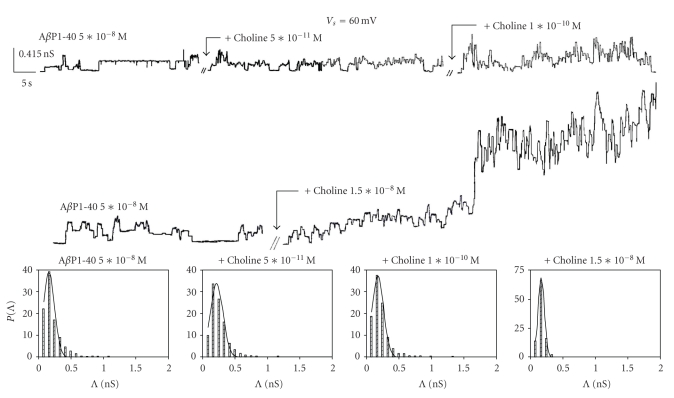
A*β*P1-40 channel activity in OxCh PLM in the absence and in the presence of different choline concentrations. Experiments were performed on an OxCh PLM in the presence of different concentrations of choline added or in the presence of high choline concentrations on the *cis*-side of the medium; the applied voltage was set at 60 mV; the arrows indicate the choline additions when the A*β*P1-40 channel fluctuates in the open state. The channel central conductance (nS ± SE)/frequency (events/min ± SD) without and with choline added to the medium was: A*β*P1-40 = 0.161 ± 0.008/7.73 ± 0.34; A*β*P1-40 + choline (5 × 10^−11^ M) = 0.215 ± 0.007/15.97 ± 0.53; A*β*P1-40 + choline (1 × 10^−10^ M) = 0.179 ± 0.004/10.56 ± 0.40; A*β*P1-40 + choline (1.5× 10^−8^ M) = 0.168 ± 0.001/11.85 ± 0.60. The number of channel considered was: A*β*P1-40 = 505; A*β*P1-40 + choline (5× 10^−11^ M) = 898; A*β*P1-40 + choline (1 × 10^−10^ M) = 652; A*β*P1-40 + choline (1.5 × 10^−8^ M) = 358. Amplitude histograms of channel conductance for each trace are shown below traces.

Figure 4 shows typical examples of single-channel chart recordings of A*β*P1-40 channel formation with associated conductance distribution histograms in OxCh PLMs without and with successive additions of choline to the *cis*-side of the medium facing the membrane or when choline in a ratio of about 1: 3 to the peptide (1.5 × 10^−8^ M) was added when the channel was fluctuating in the open state. It can be seen that single-channel activity sometimes occurred in highly variable steps, yet the frequency of channels increases by increasing the choline concentration; we also observed alternating periods of paroxystic channel activity, during which it is impossible to make a rigorous analysis of the number of channels, followed by quiescent periods that are more frequents at higher choline concentration and often followed by membrane destabilization until rupture. Furthermore, a *t*-test showed that the central channel conductance of A*β*P1-40 is statistically increased when choline at different concentrations is added to the cis-side (see capture of Figure 4). Yet, the same result was found when choline at different concentrations was added to the *trans *side (data not shown).

**Figure 5:**
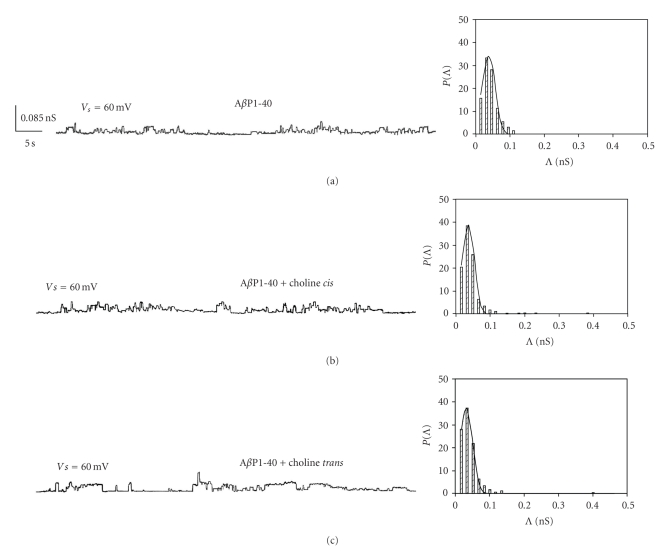
A*β*P1-40 channel activity in POPC:Ch PLM. Representative A*β*P1-40 channel in the absence (a) or in the presence of choline (5 × 10^−11 ^M) on the *cis* (b) or on the *trans* (c) side of the medium facing a POPC:Ch (65 : 35, w/w) PLM at an applied voltage of 60 mV. Amplitude histograms of channel conductance for each trace are shown next to their respective traces.

**Figure 6:**
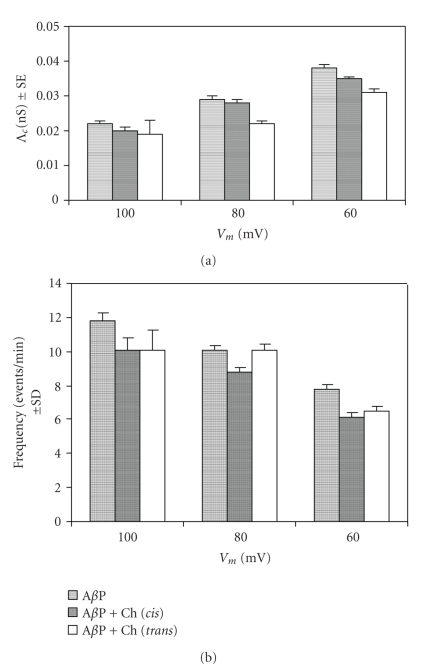
A*β*P1-40 channel mean conductance and frequency in POPC:Ch PLM. (a) Λ_*c*_ ± SE and (b) frequency ± SD of A*β*P1-40 channel without and with choline (5 × 10^−11^ M) (added to the *cis *or* trans* side of the chamber) in POPC:Ch (65 : 35, w/w) PLM. The minimum and maximum number of channels considered (*N*) out of a total number of channels considered (*N*_*t*_) was: A*β*P1-40, 761 < *N* < 1283, *N*_*t*_ = 2928; A*β*P1-40 + choline *cis, *449 < *N* < 1320, *N*_*t*_ = 2305; A*β*P1-40 + choline *trans*, 170 < *N* < 624, *N*_*t*_ = 1269.

Figure 5 shows examples of chart recordings of A*β*P1-40 channel formation in POPC:Ch PLMs. In these PLMs, no matter if choline is added to the *cis* or the *trans* side of the membrane, it does not increase A*β*P1-40 ion channel activity or conductance, nor does it modify the voltage dependence and the Λ_*c*_ values of the A*β*P1-40 channel (Figure 6(a)). The Λ_*c*_ values of the A*β*P1-40 channel in POPC : Ch PLMs are lower than those in OxCh PLMs.

Our results indicate that the presence of choline (on the *cis* or *trans* side) does not modify the voltage dependence of A*β*P1-40 channels in OxCh or POPC : OxCh PLMs (Figures 3(a) and 6(a)).

### 3.2. Effect of Choline on A*β*P1-40 Channel Frequency and Lifetime

The A*β*P1-40 frequency values are generally higher at positive than those at negative applied voltages in OxCh PLMs (Figure 3(b)). Adding choline (5 × 10^−11^ M) to the *cis* or *trans* side doubled and trebled, respectively, the mean frequency values of the A*β*P1-40 channel at positive applied voltages. On the other hand, at negative applied voltages, choline exerts no effect on A*β*P1-40 channel frequency, either when present on the *cis* or on the *trans* side of the medium facing the membrane. The frequency increases as a function of choline concentration, and this increase occurs early. In fact, the frequency of A*β*P1-40 channel was: 7.73 ± 0.34 without choline, 15.97 ± 0.53 for a choline concentration of 5 × 10^−11^ M, 10.56 ± 0.40 for a choline concentration of 1 × 10^−10^ M, and 11.85 ± 0.60 for a choline concentration of 1.5 × 10^−18^ M. It is worth noting that these last two values are underestimated owing to the paroxystic activity (see Figure 4). In POPC : Ch PLMs, A*β*P1-40 channel frequency is not sensitive to the presence of choline on the *cis* or *trans* side of the membrane (Figure 6(b)).

Another parameter used to characterize a channel is its lifetime. Single-channel current recordings with a conspicuous number of channels were analysed to obtain cumulative open-state lifetime distributions that are reported for the different experimental conditions. In OxCh PLMs, analysis of the open-time distributions for A*β*P1-40 single channels is reported in Table 1, where the functions with statistically significant better description are indicated. It can be noted that at positive applied voltages the fast channel lifetime component prevails (*P* < .05), whereas at negative applied voltages the channel manifests both the fast and slow components of lifetime (*P* < .05), except for at −20 mV where it does not clearly distinguish between single and double exponentials (*P* = .102).

When choline is on the *cis* or *trans* side, the results of the open time distribution analysis for all positive and for low negative applied voltages indicate a statistically significant better description (*P* < .05) for two-exponential functions; except for −40 and −60 mV (choline* cis*) and −60 mV (choline *trans*) where there is a statistically significant better description (*P* < .05) for one-exponential functions, however the values of *τ*_1_ in the presence of choline are higher than that of A*β*P1-40. The prevalence of dual channel populations or the higher values of *τ*_1_ clearly indicates that choline (either *cis* or *trans*) seems to stabilize the A*β*P1-40 single channel. 

In POPC : Ch PLMs, analysis of the open-time distribution for A*β*P1-40 single channels does not clearly distinguish between single and double exponentials (at applied voltages in this study) (*P* ≥ .078). When choline is on the *cis* side, the results of open-time distribution analysis indicate a statistically significant better description (*P* < .05) for one-exponential functions, except at applied voltages of 100 mV where it does not clearly distinguish between single and double exponentials (*P* = .39). When choline is on the *trans* side, the results of open time distribution analysis give a statistically significant better description (*P* < .05) for a one-exponential function at an applied voltage of 60 mV, whereas it does not clearly distinguish between single and double exponentials at an applied voltage of 80 mV (*P* = .37). At an applied voltage of 100 mV, the number of channels is not conspicuous enough to provide a reliable analysis of open-time distribution (Table 2). In any case, the addition of choline, either on the *cis* or the *trans* side, does not seem to modify the lifetime and stability of the ion channel.

### 3.3. Ion Selectivity

The ion selectivity of A*β*P channels in OxCh or POPC:Ch membranes in the presence of choline in the *cis* or *trans* medium was determined by means of reversal potential and *I*-*V* relationship at different transmembrane potentials under asymmetrical solution conditions (see methods).

In OxCh PLMs, the *P*_*K*_^+^/*P*_Cl_^−^ calculated by (2) was 0.50/0.38 *cis*/*trans,* respectively. 

**Figure 7:**
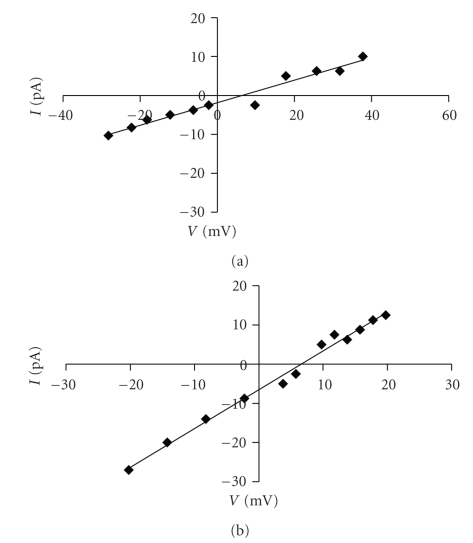
A*β*P1-40 channel ion selectivity in OxCh PLM. A*β*P1-40 channel selectivity in the presence of choline (5 × 10^−11^ M) *cis* (a) or *trans* (b) in OxCh PLM. The amplitude of the channel current (pA) is plotted as a function of the transmembrane potential (*V*). Conductance was determined by linear regression of the current values from −30/−20 mV to 40/20 mV (choline *cis*/*trans*) in asymmetrical solutions 100 mM/50 mM KCl *cis*/*trans*. Intercept (5.76/7.76 mV, choline *cis*/*trans*) was used to calculate *P*_*K*_^+^/*P*_Cl_^−^.

Approximately the same result was obtained with the *I*-*V* curve (Figure 7), where the amplitude of the channel events at each membrane potential was used. In fact, the reversal potential was 6.42 mV with choline in the *cis* chamber and 6.87 mV with choline in the *trans* chamber; the selective ratio *P*_*K*_^+^/*P*_Cl_^−^ was 0.46/0.43 *cis*/*trans*, respectively. These results seem to indicate that the ion selectivity toward anions of the A*β*P1-40 channel in OxCh PLMs [14] is not modified by the presence of choline.

The ion selectivity of the A*β*P1-40 channel in POPC:Ch PLMs remains anionic; in fact, the reversal potential was 9.76 mV and the *P*_*K*_^+^/*P*_Cl_^−^ was 0.28.

**Figure 8:**
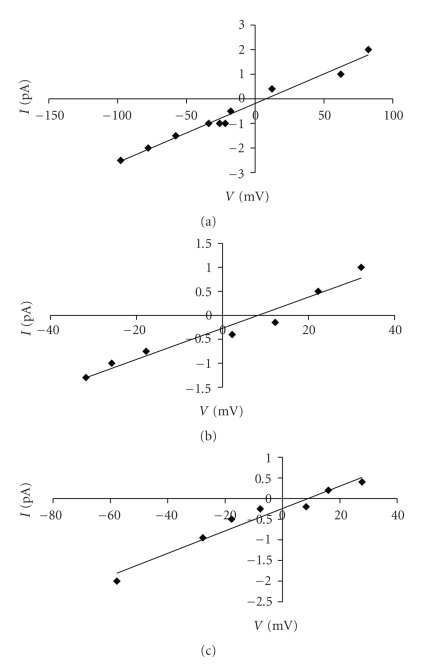
A*β*P1-40 channel ion selectivity in POPC:Ch PLM. A*β*P1-40 channel selectivity (a), in the presence of choline (5 × 10^−11^ M) *cis* (b) or *trans* (c) side in POPC:Ch (65 : 35, w/w) PLM. The amplitude of the channel current (pA) is plotted as a function of the transmembrane potential (*V*). Conductance was determined by linear regression of the current values from −150 to 100 mV (a) and from −40/−80 mV to 40/40 mV (choline *cis*/*trans*) in asymmetrical 100 mM/50 mM KCl *cis*/*trans *solutions. Intercept (9.76/5.76/7.76 mV, (a)/(b)/(c) resp.) was used to calculate P_K_^+^/P_Cl_^−^.

When choline was present on the *cis*/*tran*s side the reversal potential was 5.76/7.76 mV and the *P*_*K*_^+^/*P*_Cl_^−^ was 0.50/0.38 respectively. Approximately the same result was obtained with the *I*-*V* curve (Figure 8). In fact, the reversal potential was 8.99 mV in the absence of choline, with a selective ratio *P*_*K*_^+^/*P*_Cl_^−^ of 0.32. The reversal potential was 6.67 mV with choline in the *cis* chamber and 6.47 mV with choline in the *trans* chamber; with a selective ratio *P*_*K*_^+^/*P*_Cl_^−^ of 0.44/0.45 *cis*/*trans*, respectively.

### 3.4. Effect of Choline on A*β*P1-40 Secondary Structure

**Figure 9:**
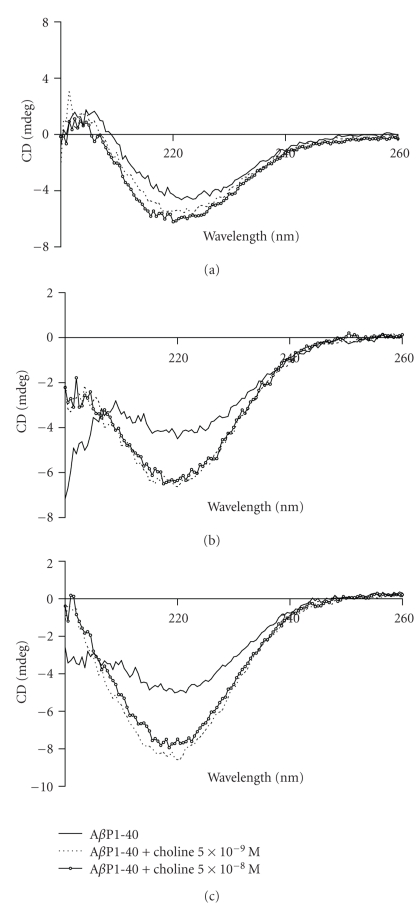
A*β*P1-40 secondary structure. Far-UV CD spectroscopy of A*β*P1-40 (5 × 10^−6^ M) in the absence and in the presence of choline (5.77 × 10^−8 ^M; 5.77 × 10^−9 ^M) after 5 minutes (a), 24 hours (b) and 48 hours (c) of incubation. See text for experimental conditions.

To test whether choline modifies the secondary structure of A*β*P1-40, we carried out CD experiments using A*β*P1-40 samples in the absence or in the presence of choline in a molar ratio of 1 : 100 and 1 : 1000 choline : A*β*P.  Figure 9 shows the CD spectra of A*β*P1-40 without and with choline measured after 5 minutes (T0), 24 hours (T24), and 48 hours (T48). 

The CD spectra qualitatively indicate that A*β*P1-40 conformation in an aqueous environment is predominantly *β*-sheet and random coil, while the *α*-helical content is very small. Our results are in line with other authors' findings [11]. The features of the spectra show that choline at the two concentrations used does not modify the secondary structure of the peptide. Moreover, it increases the signal intensity after 24 and 48 hours of incubation. This could mean that choline stabilizes the A*β*P1-40 structure, counteracting peptide aggregation.

### 3.5. Choline Permeability across Undoped and Doped OxCh Membranes

Control experiments were designed to test the effect of ethanol on membrane integrity. The addition of 4 *μ*l of ethanol to both sides of the OxCh membrane, under stirring, does not influence the capacitance (0.32 *μ*F/cm^2^) and the conductance (25 pS) of the membrane over a long period of time (about 6 hours).

The permeability coefficients of [^3^H]choline across the OxCh membrane, undoped and doped with A*β*P1-40, were: 47.1 ± 2.14 and 43.8 ± 7.9 (cm × sec^−1^ × 10^−6^), respectively.

This data indicates that choline, despite showing a high permeability coefficient across OxCh membranes, is unable to cross the A*β*P1-40 channel.

## 4. Discussion

Early evidence showed that A*β*P1-40 is initially deposited on the membranes and acts as a “seed” for the formation of fibrils [19], although the involvement of A*β*P fibrillation *in vivo* in the process of neurodegeneration and the development of AD is not an irrefutable criterion for defining the pathology. Recent studies have shown that A*β*P induces toxicity on cholinergic neurons *in vitro* and *in vivo* by increasing, via distinct mechanisms, Ach turnover. In neuronal cells, choline is a precursor of ACh, but also a product of ACh hydrolysis after the neurotransmission process. The mechanisms involved in cholinergic dysfunction are under intense investigation because of their potential therapeutical implications [20]. Although the basal extracellular choline concentration is higher (about 5 *μ*M) [21] as compared to the concentration used in the present study, the sensitivity of our experimental system, suitable for high-resolution ion current recordings, does not allow us to use choline concentration higher than 1.5 × 10^−8^ M. In fact, the activation of A*β*P channels by choline at concentration higher than 1.5 × 10^−8^ M generates an electrical signal that is so large that it exceeds the maximum level of detection by the recording system furthermore, these conditions will lead to membrane instability eventually resulting in membrane breakage.

The human brain contains as much as 25% of the total pool of Ch, mainly concentrated in the myelin sheath. However, there are also considerable amounts of Ch in neuronal plasmalemma and in lipid rafts. One interesting aspect of the Ch molecule seems to be its affinity for many proteins such as porins [22, 23], for peptides such as magainin-2 [24], A*β*P1-40 and A*β*P1-42 [14], and for peptides associated with myelin; examples of Ch-dependent proteins are prominin, synaptophysin, platelet-derived growth factor receptor, hemolysin, acetylcholine-receptor, peripheral myelin protein 22, and prions [25–31]. In particular, it has been demonstrated that Ch at a concentration of 33% in model membranes and in vesicles can favour the transition of A*β*P1-40 and human cytoplasmic domain of myelin protein zero into alpha helix [32]. On the other hand, membrane Ch induces the structural conformation of many peptides for incorporation. 

Results in OxCh and POPC : Ch planar lipid membranes (PLMs) confirm our previous findings [14] and further indicate the role played by membrane composition in the alpha-helix structure induction mechanism needed for A*β*P incorporation into membranes. Furthermore, we found sensitivity to choline addition of A*β*P channels incorporated into OxCh PLMs; indeed, choline does not modify some channel characteristics, such as voltage sensitivity, voltage-dependence, and selectivity, but increases channel conductance, frequency, and stability, as indicated by the presence of two channel life-time components. 

In the Type I A*β*P1-40 channel model proposed by Durell et al. [33], the amino acids able to form hydrogen bonds could be R5, D7, E11, K16, and D23. This has been invoked to explain the cation selectivity of the A*β*P1-40 channel. The A*β*P1-40 channel incorporated in our membranes is anion selective. Choline does not modify the anion selectivity of A*β*P channel incorporated in OxCh membrane, the aggregated configuration of A*β*P peptide could be different, although this channel shows the same diameter, as reported in our previous paper [14], found in the theoretical calculation of the model proposed by Durell et al. [33].

Incorporation in POPC PLMs containing 33% Ch seems to be driven by hydrophobic interaction, as the high hydrophobic amino acid content in the C-terminus of A*β*P1-40 would suggest. Furthermore, our results seem to indicate that the A*β*P1-40 channel is smaller than that formed in OxCh membranes, which could reflect a lower number of A*β*P1-40 molecules assembled to form ion channels. However, it is worth noting that in POPC : Ch PLMs, the channel maintains its anionic selectivity, thus pushing the membrane into a hyperpolarized state. 

Our CD results, obtained in an aqueous environment, indicate that choline does not modify A*β*P1-40 peptide conformation but rather stabilizes it in a random coil conformation that is less prone to fibrillate. This result is consistent with the notion that the role played by choline is to counteract peptide aggregation, by maintaining it in the stabilized random coil form, so that in the membrane alpha-helices will arise in the peptide that tend to pack together to form ion channel rather than remain dispersed. Hydrogen bonds and ion pairs can be used to drive the association of local regions in the helices [34]. It is interesting to note that Kar et al. [9] found a concentration-dependent inhibition of high-affinity choline uptake by A*β*P in rat hippocampal slides, indicating a direct interaction between A*β*P and choline. A*β*P1-40 present on the extracellular side of PC12 cells as a monomer has been found to increase the conductance of choline trapped inside cells. The authors [35] suggest that A*β*P1-40 peptide acts as a choline carrier or that it modifies an endogenous ion channel or transporter. However, a suggestive possibility could be that choline crosses the A*β*P1-40 channel. To verify this possibility, permeability experiments on OxCh PLMs have been carried out. In our experimental conditions, where there are no other cellular components except for the A*β*P1-40 channel, choline does not cross the A*β*P1-40 channel. 

Study of the effects of ACh on A*β*P1-40 by means of CD indicates that ACh has no effect on peptides in solution; moreover, single channel conductance decreases when ACh is added on the *cis* side when the channel is open; on the other hand, in the same experimental conditions, ACh addition on the *trans* side did not modify channel conductance [36]. This indicates a specific effect of choline on A*β*P1-40.

## 5. Conclusions

Our present findings support and extend the emerging concept that cholesterol can easily incorporate A*β*P1-40, thus favouring its clearance; therefore, by removing the peptide from the environment, fibrillation will be avoided. Furthermore, the anionic nature of the A*β*P1-40 channel formed in cholesterol-containing PLMs could be protective for the cells. Choline specifically accelerates this process. The greater effect of choline on depolarized membrane states found in this work could be of relevance for synaptic activity in that the higher ionic conductance, frequency and lifetime, as well as the anionic selectivity, all contribute to returning the membrane potential to basal condition. This could exert a kind of modulation on the synapses. 

It may be speculated that owing to the high choline permeability found across the OxCh membrane, and the *in vivo* facilitated/active choline transport, ACh synthesis takes place quickly. The increase in ACh delivery and/or ACh esterase modulation may be beneficial to synaptic activity. Obviously, these aspects are merely speculative because we do not have any evidence that this occurs in *vivo.*

## Figures and Tables

**Table 1:** The channel lifetime of A*β*P1-40 in OxCh PLM. The fitted lifetime (see text) of A*β*P1-40 in the absence (a) and in the presence of choline (5 × 10^−11^ M) added on the *cis*/*trans* side of the medium facing the OxCh PLM (b/c, resp.). The minimum and maximum number of channels considered (*N*) out of a total number of channels considered (*N*_*t*_) was: (a) 125 < *N* < 503, *N*_*t*_ = 1214; (b) 95 < *N* < 217, *N*_*t*_ = 834; (c) 102 < *N* < 186, *N*_*t*_ = 857. *F*-test, *P = .102.

Vs (mV)	a		b		c	
	*τ*_1_ (s)	*τ*_2_ (s)	*τ*_1_ (s)	*τ*_2_ (s)	*τ*_1_ (s)	*τ*_2_ (s)
60	2.07		1.53	8.34	1.45	7.76
40	3.56		1.88	19.61	1.58	8.46
20	1.12		1.29	20.01	1.17	6.49
−20	1.05*		2.23	32.26	0.25	5.03
−40	1.17	4.96	3.69		0.15	3.94
−60	1.33	8.99	2.23		2.54	

**Table 2:** The channel lifetime of A*β*P1-40 in POPC:Ch PLM. The fitted lifetime (see text) of A*β*P1-40 (a), A*β*P1-40 and choline (5 × 10^−11^ M) added to the *cis*/*trans* side of the chamber (b/c, resp.) channels in the POPC:Ch membrane. The minimum and maximum number of channels considered (*N*) out of a total number of channels considered (*N*_*t*_) was: (a) 94 < *N* < 240, *N*_*t*_ = 448; (b) 120 < *N* < 196, *N*_*t*_ = 435; (c) 19 < *N* < 111, *N*_*t*_ = 205. *F*-test, *P ≥ .078; **P = .39; ***P = 0.37.

Vs (mV)	a	b	c
	*τ*_1_* (s)	*τ*_1_ (s)	*τ*_1_ (s)
100	2.50	1.72**	—
80	3.13	3.03	2.99***
60	3.25	2.32	3.11
